# A Mouse Model for Studying the Clearance of Hepatitis B Virus In Vivo Using a Luciferase Reporter

**DOI:** 10.1371/journal.pone.0060005

**Published:** 2013-04-05

**Authors:** Sheng-qiang Liang, Juan Du, Hu Yan, Qian-qian Zhou, Yong Zhou, Zhen-nan Yuan, Shao-duo Yan, Qiu-xia Fu, Xiao-hui Wang, Shuai-zheng Jia, Jian-chun Peng, Yang-gen Zhang, Lin-sheng Zhan

**Affiliations:** 1 Lab of Blood-Borne Viruses, Beijing Institute of Transfusion Medicine, Beijing, China; 2 Department of Clinical Laboratory, the 175th Hospital of PLA, Affiliated Dong nan Hospital of Xiamen University, Zhang zhou, China; Yonsei University, Republic of Korea

## Abstract

Hepatitis B virus(HBV) infection remains a global problem, despite the effectiveness of the Hepatitis B vaccine in preventing infection. The resolution of Hepatitis B virus infection has been believed to be attributable to virus-specific immunity. In vivo direct evaluation of anti-HBV immunity in the liver is currently not possible. We have developed a new assay system that detects HBV clearance in the liver after the hydrodynamic transfer of a reporter gene and over-length, linear HBV DNA into hepatocytes, followed by bioluminescence imaging of the reporter gene (Fluc). We employed bioluminescence detection of luciferase expression in HBV-infected hepatocytes to measure the Hepatitis B core antigen (HBcAg)-specific immune responses directed against these infected hepatocytes. Only HBcAg-immunized, but not mock-treated, animals decreased the amounts of luciferase expression, HBsAg and viral DNA from the liver at day 28 after hydrodynamic infection with over-length HBV DNA, indicating that control of luciferase expression correlates with viral clearance from infected hepatocytes.

## Introduction

Hepatitis B virus (HBV) is a hepatotropic, noncytopathic virus that causes acute and chronic liver diseases and, subsequently, hepatic cirrhosis and hepatocellular carcinoma. Although an effective preventive vaccine is available, there are currently approximately 300 million people who are chronically infected [1]. Worldwide, the number of deaths from liver cancer caused by HBV infection likely exceeds one million per year. Although an effective vaccine was introduced more than 20 years ago and has shown great success, existing HBV carriers account for up to 20% of the population in certain Asian and African countries [2], and unfortunately, vaccination is not a treatment for established infections.

It has been established that the host immune response plays a major role in the outcome of HBV infection. During acute HBV infection, the development of a strong cellular immune response, directed to multiple viral Ags, is associated with the resolution of HBV infection and lifelong antiviral immunity. However, in chronically infected patients, these responses are markedly attenuated [3]–[6]. Several animal models have been used to study HBV pathogenesis and the immune response to HBV, including chimpanzees [7], the woodchuck [8], a transgenic mouse [9], and HBV-transfected mice through the delivery of HBV genomes into murine hepatocytes in vivo using either adenoviral gene transfer (AdHBV) or hydrodynamic injection (HDI-HBV) [10], [11]. However, antiviral immunity in the liver is determined only by indirect measures such as serum ALT levels, histochemistry or a reduction in serum levels of viral proteins or genes. In the present study, we describe the development of a nontransgenic model consisting of HBV 1.2 full-length DNA, with the HBV core promoter controlling the expression of the firefly luciferase gene, and the use of this model for monitoring viral clearance in vivo using bioluminescence imaging, similarly to a study by Stabenow et al. [12]. This technique, which has been used to study a variety of bacterial and viral infections [13]–[15], offers significant advantages over conventional pathogenesis studies, because this technique can 1) be used to quantitatively visualize viral infections in living animals and 2) allow disease progression and outcome to be directly linked to viral replication and viral load.

## Materials and Methods

### Ethics Statement

All animal studies were carried out according to the guidelines established by the ethics committee of the National Beijing Center for Drug Safety Evaluation and Research (Permit No. 11–1725). The mice were anesthetized with ketamine and xylazine for the procedures, including the intramuscular injections, in vivo electroporation, and hydrodynamic injections. The mice were sacrificed by ether anesthesia.

### Animals

C57BL/6 and BALB/c mice (males, 4–6 weeks old) were purchased from the National Beijing Center for Drug Safety Evaluation and Research and housed in cages in a controlled environment (22–25°C, 50% humidity, 12 h light/dark cycle).

### Plasmid Construction

HBV 1.2 full-length DNA was subcloned from the plasmid pAAV/HBV1.2, which was kindly provided by Pei-Jer Chen (National Taiwan University College of Medicine, Taipei, Taiwan), encoding an HBV fragment longer than the full length of the HBV genome [11], [16]. This fragment was subcloned into the *Xho*I/*Mlu*I site downstream of the firefly luciferase gene in the pGL3-CP-Fluc vector [17] to produce pGL3/Fluc-HBV1.2 (Fig. S1). Plasmid pGL3-HBV1.2 was generated by deleting the Fluc gene from the pGL3/Fluc-HBV1.2 plasmid between the NcoI and XbaI sites. For the generation of pVAX1-HBc, the HBV core sequence was amplified by PCR using pAAV/HBV1.2 as the template and was cloned into the *EcoR*I/*BamH*I site of the pVAX1 vector (Invitrogen, Carlsbad, CA). All constructs were sequenced to confirm their identities and insert orientations.

### Cell Cultures, DNA Transfections, and Luciferase Assays

Hepa1-6 and HuH-7 cells were purchased from ATCC (Manassas, VA) and cultured in DMEM (Gibco, Carlsbad, CA) supplemented with 10% FBS (HyClone, South Logan, UT) at 37°C in 5% CO_2_/air. Transient transfections were performed using Lipofectamine 2000 (Invitrogen, Carlsbad, CA), starting with approximately 1×10^5^ cells/well in 24-well dishes with 0.6 µg of plasmid DNA mixture, following the manufacturer’s instructions. Plasmids were purified with a Qiagen Plasmid Purification Kit (Hilden, Germany). For each well, 1 ng of a plasmid encoding the *Renilla* luciferase gene driven by the herpes simplex virus thymidine kinase (HSV-TK) promoter (pRL-TK, Promega) was included to monitor transfection efficiency. After 48 h, the cells were washed with PBS and harvested in 100 µl of Passive Lysis Buffer (PLB, Promega). *Firefly* and *Renilla* luciferase activity were both measured in a GloMax™ 96 Luminometer from 20 µl of lysate using the Dual-Luciferase Reporter Assay System (Promega).

### In vivo Gene Delivery and Determination of Luciferase Expression in the Mouse Liver

C57BL/6 and BALB/c mice (males, 4–6 weeks old, from the breeding colonies of the National Beijing Center for Drug Safety Evaluation and Research) were anesthetized with ketamine and xylazine. Ten micrograms of DNA was injected into the tail veins of mice in a volume of saline equivalent to 8% of the mouse body weight in a time range of 5 to 8 s [18], [19]. Animals were imaged in the Xenogen IVIS-50 optical imaging system at the indicated times described in the article.

### Detection of HBV Antigen, Antibody, and Serum Alanine Aminotransferase

Serum levels of HBsAg in the mice were determined with the PRISM System Kit (Abbott) or commercially available ELISA kits (InTec Products, INC., Xiamen), and the reporting unit is IU/ml or S/CO ratio. Serum alanine aminotransferase was measured in 25 µl samples using a microtiter plate assay (Pointe Scientific, Inc., Canton, MI).

### Immunohistochemical Staining for HBcAg

Liver tissues were collected from mice killed at the indicated time points and were fixed in 10% formaldehyde, embedded with paraffin and cut into 4 µm thick sections. For intrahepatic HBcAg detection, the sections were incubated with rabbit anti-HBc antibody (Rockland Immunochemicals, Inc.) and were detected by DAB staining. The liver sections were also stained with hematoxylin.

### Detection of Serum HBV DNA

Serum samples were collected at the indicated time points after hydrodynamic injection of pGL3/Fluc-HBV1.2. The total DNA of the serum samples was extracted and tested for the presence of HBV DNA by real-time PCR.

### Immunization of Mice with Recombinant Proteins or Plasmids

Mice used for studying the effects of preexisting HBcAg-specific immunity were injected intramuscularly in the tibialis anterior muscle with 100 µg of pVAX1-HBc or pVAX1 dissolved in 50 µl of PBS, thrice within a 2-week interval. Both injections were followed by *in vivo* electroporation to increase the expression levels of the injected plasmids [20], [21]. For the protein immunizations, mice were injected subcutaneously (s.c.) in the back with 2 µg of rHBsAg (GlaxoSmithKline Biologicals S.A.), thrice within a 2-week interval.

### Analysis of T-Cell Function Ex Vivo

Livers and spleens were harvested from immunized mice at 21 dpi. Cell suspensions of each liver were resuspended in 4 mL of 40% Percoll (GE Healthcare, Munich, Germany) and underlaid with 4 mL of 70% Percoll. After centrifugation for 20 minutes at 1400×g at room temperature, liver-associated lymphocytes were collected from the interface between the 40% and 70% Percoll layers. Cell suspensions of each spleen were resuspended in 4 mL of 40% Percoll (GE Healthcare, Munich, Germany) and underlaid with 4 mL of 70% Percoll. After centrifugation for 20 minutes at 1400×g at room temperature, spleen-associated lymphocytes were collected from the interface between the 40% and 70% layers. Lymphocytes (5×10^5^) were cultured in RPMI medium 1640 (Invitrogen) with 8% fetal calf serum in the presence of 10 µmol/L peptides, HBc93-100 and HBs190–197, respectively, for 5 hours. For intracellular interferon-γstaining, monensin (0.1%; e-Bioscience) was added to the culture 1 hour after the culture had been set up. The cells were harvested for CD8α surface staining, subjected to intracellular staining of interferon-γ using PE-Cy-7 conjugated anti-IFN-γ antibodies (clone XMG1.2; BD Pharmingen), cell fixation/permeabilization using a kit (BD Biosciences, Heidelberg, Germany), and flow cytometric analysis.

### Histology

For routine histological analysis, formalin-fixed paraffin-embedded liver samples were cut into sections 4 µm thick, deparaffinized in xylene, and dehydrated through a series of decreasing concentrations of ethanol. Sections were stained with hematoxylin and eosin.

### Statistics

All data were reported as the mean±SD. For statistical comparisons, parametrical data were compared using a Student’s t-test. Differences were considered significant at P<0.05.

## Results

### Hydrodynamic Injection of pGL3/Fluc-HBV1.2 Leads to Reporter Gene and HBV Persistence in vivo

**Figure 1:**
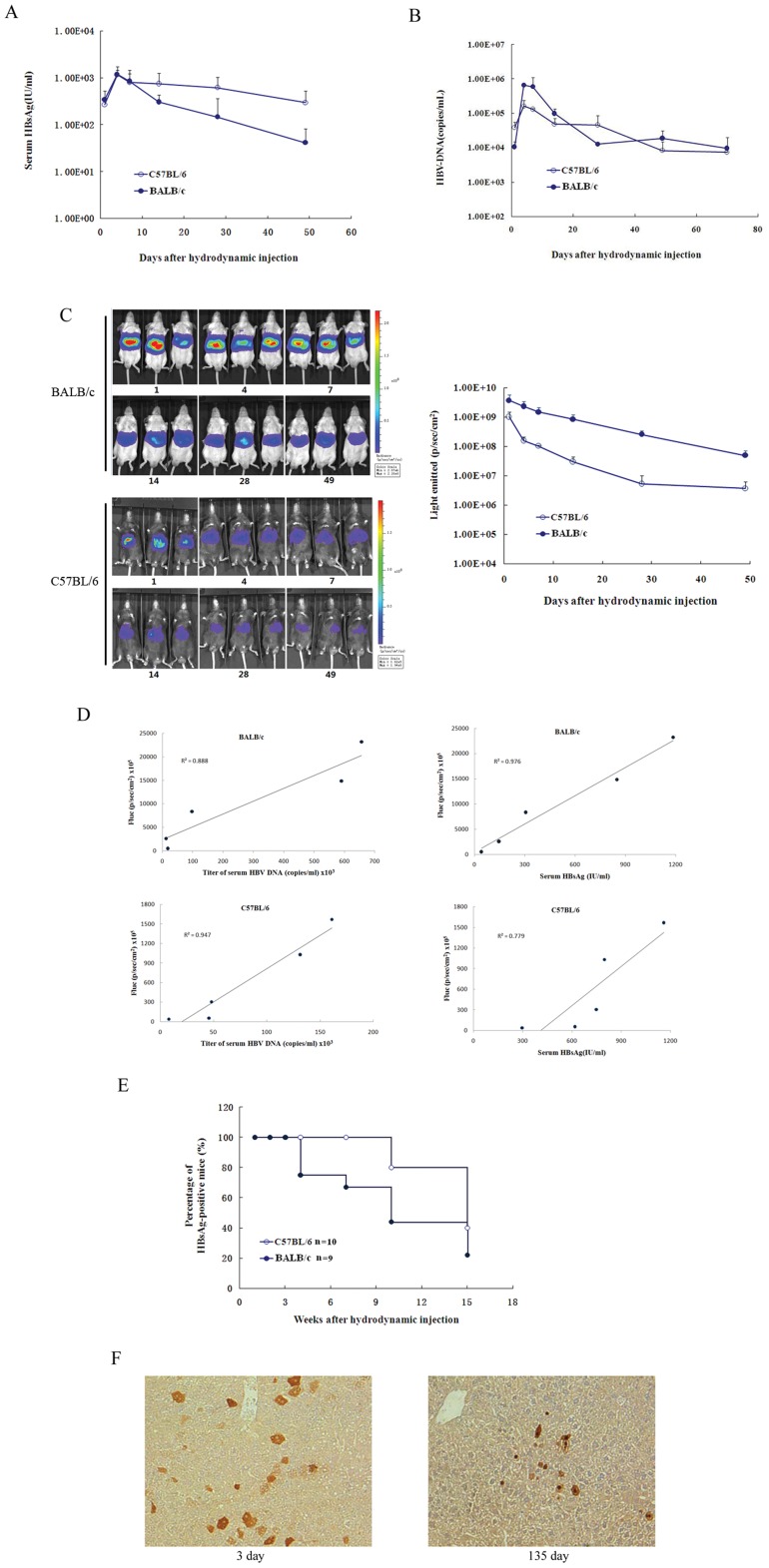
HBV persistence in mice induced by hydrodynamic injection of pGL3/Fluc-HBV1.2. A. Titer of serum HBsAg in C57BL/6 or BALB/c mice after HBV plasmid injection. The detection limitation is 0.05 IU/ml. B. Serum HBV DNA levels in C57BL/6 or BALB/c mice after HBV plasmid injection. The detection limitation is 100 copies/ml. C. Bioluminescence image of C57BL/6 (H-2b) or BALB/c (H-2d) mice after hydrodynamic injection. D. Fluc expression in the liver and both serum HBsAg and HBV DNA levels showed strong linear correlation. E. Immunohistochemical staining for HBcAg in hepatocytes of HBsAg-positive C57BL/6 mice (magnification, 200x). In C57BL/6 mice, HBcAg-positive cells were maintained at stable levels on day 3 and day 135. F. Positive rate of serum HBsAg in C57BL/6 (*n* = 10) or BALB/c (*n* = 9) mice receiving injection with pGL3/Fluc-HBV1.2 at different time points after injection.

Chen and colleagues have shown that a single hydrodynamic injection of a replication-competent HBV DNA, pAAV/HBV1.2, into mice could result in HBV persistence for greater than 1 year in a significant proportion of recipients [11]. Thus, the HBV DNA sequences in pAAV/HBV1.2 were cloned into a different vector, pGL3-CP-Fluc, to construct pGL3/Fluc-HBV1.2, encoding the HBV genes and a reporter gene (Fluc). To determine whether these constructs could express both the HBV gene and Fluc, pGL3-HBV1.2, pGL3-CP-Fluc and pGL3/Fluc-HBV1.2 were transfected into HuH-7 cells. Forty-eight hours after the transfections, Fluc activity and HBsAg were measured. Although Fluc activity was lower in cells transfected with pGL3/Fluc-HBV1.2 than in those transfected with pGL3-CP-Fluc, HBsAg levels were lower in cells transfected with pGL3/Fluc-HBV1.2 than in those transfected with pGL3 -HBV1.2 (Fig. S2). We verified that pGL3/Fluc-HBV1.2 could sustain HBV gene and reporter gene expression in cells. Next, we investigated whether these constructs could express HBV genes and Fluc in the mouse liver. Plasmid pGL3/Fluc-HBV1.2 was injected hydrodynamically into the tail veins of C57BL/6 (H-2^b^) or BALB/c (H-2^d^) mice. After the injection, the mice were regularly bled to monitor the serum levels of HBsAg and HBV DNA. In the sera of BALB/c and C57BL/6 mice, HBsAg accumulated to an average concentration of 1.16×10^3^ and 1.19×10^3^ IU per milliliter, respectively, on day 4 postinjection (dpi) (Fig. 1A). After this peak, levels of HBsAg in the serum of the BALB/c mice dropped by nearly two orders of magnitude, reaching a concentration of 4.86×10^1^ IU per milliliter by day 49. In C57BL/6 mice, HBsAg levels declined much more slowly and fell to 2.97×10^2^ IU per milliliter on day 49. The levels of serum HBV DNA were quantified using real-time PCR. In BALB/c and C57BL/6 mice, the average titer of serum HBV DNA was 1.06×10^4^ and 3.89×10^4^ copies per milliliter at 1 day postinjection (dpi) and peaked at 6.57×10^5^ and 1.61×10^5^ copies per milliliter, respectively, during 4–7 dpi (Fig. 1B). In BALB/c mice, viremia subsequently declined with logarithmic kinetics through day 7 (5.91×10^5^ copies per ml) and day 28 (1.27×10^4^ copies per ml). In contrast, viremia declined moderately in the C57BL/6 mice after 7 dpi. The average titers of serum HBV DNA in BALB/c mice were similar to those of the C57BL/6 mice.

Bioluminescence imaging was performed to examine Fluc activity at different time points after DNA injection. As illustrated in Fig. 1C, Fluc activity was significantly high in all of the mice at 1 dpi. After 1 dpi, the Fluc activity declined slowly and remained at a stable level until 49 dpi. In the BALB/c mice, Fluc activity was greater, at approximately one order of magnitude greater than that in C57BL/6 mice throughout the length of the experiment.

For the Fluc reporter gene to be used for monitoring HBsAg and titers of HBV DNA in serum, it was essential to demonstrate a correlation between the expression of the reporter gene and serum HBsAg or HBV DNA. A strong positive linear correlation was present between Fluc expression in the liver and both serum HBsAg and HBV DNA levels on days 4 to 49 (r = 0.988 and 0.942, respectively, for BALB/c mice and r = 0.883 and 0.973, respectively, for C57BL/6 mice) (Fig. 1D). These data demonstrate the ability to use reporter gene expression as a measure of serum HBsAg and titers of serum HBV DNA.

We continued to monitor serum HBsAg in BALB/c and C57BL/6 mice receiving pGL3/Fluc-HBV1.2. In approximately 80% of the C57BL/6 mice, serum HBsAg persisted for 10 weeks, whereas only 40% of the BALB/c mice remained HBsAg positive at 10 weeks postinjection (Fig. 1E). These results demonstrated that the host genetic background influences HBV persistence in the mouse liver, a finding that had been previously reported by Chen Pei-Jer et al. [11]. Liver tissues were collected from serum HBsAg-positive and HBsAg-negative C57BL/6 mice at 3 and 135 dpi and were assayed for levels of HBcAg (Fig. 1F). At 3 dpi, HBsAg-positive C57BL/6 mice expressed high levels of HBcAg, and HBcAg-positive hepatocytes were randomly distributed throughout the liver lobule. At 135 dpi, HBcAg-positive hepatocytes were rare. Both cytoplasmic and nucleic HBcAg were also detected in the livers of HBsAg-positive mice at two different time points.

### In vivo Bioluminescence Imaging Detects HBcAg-specific Immunity and Clearance HBV

**Figure 2:**
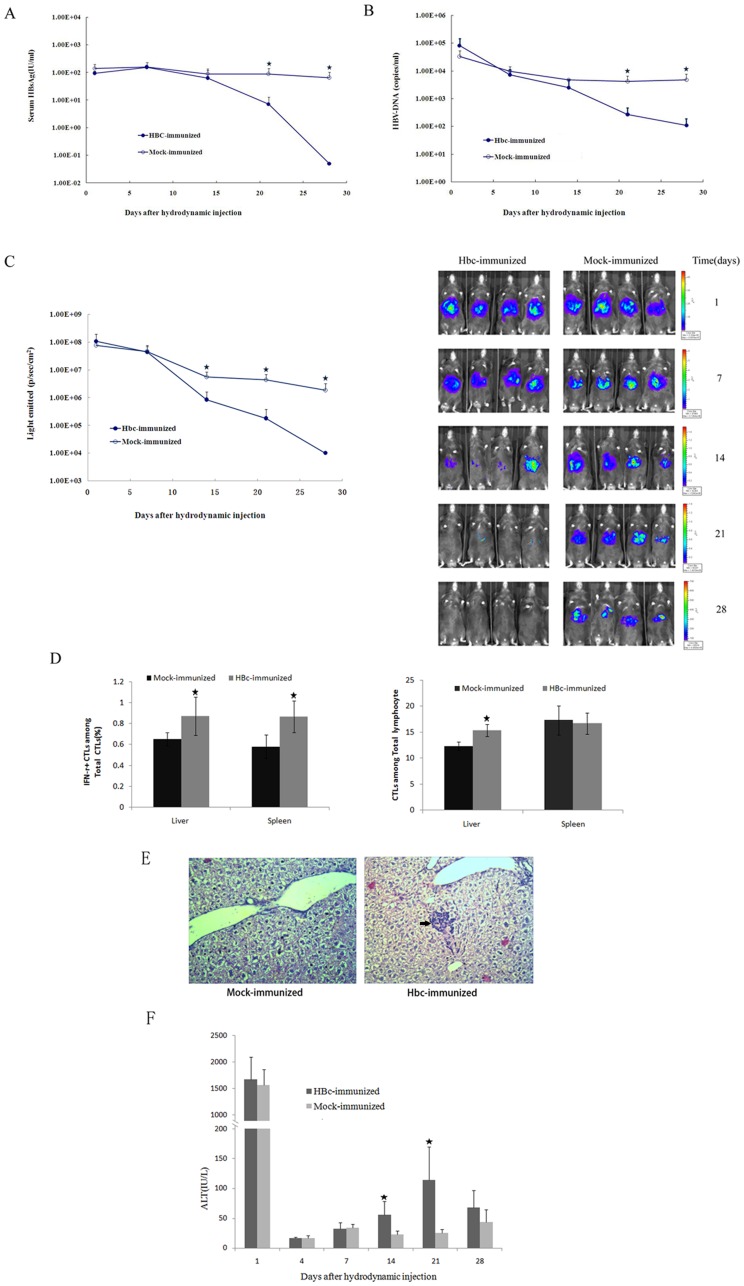
Monitoring anti-HBV CD8^+^ T cell function in the liver by bioluminescence imaging after immunization. A. Titers of serum HBsAg in HBcAg- or mock-immunized C57BL/6 mice after hydrodynamic injection of pGL3/Fluc-HBV1.2. B. Serum HBV DNA in the HBcAg- or mock-immunized C57BL/6 mice after hydrodynamic injection. The detection limit for HBV DNA in our system was 100 copies/ml. C. Real-time in vivo imaging of Fluc expression in the HBcAg- or mock-immunized C57BL/6 mice over a period of 28 days after hydrodynamic injection. D. Liver- and spleen-associated CTLs were stimulated ex vivo with HBV peptides for 5 hours. *Left*: percentages of interferon-γ–secreting CTLs on day 21 postinfection (n = 4). *Right*: percentages of CTLs on day 21 postinfection (n = 4). Statistical differences are indicated as follows: * = p<0.05. E. Hematoxylin/eosin staining of liver sections from mice treated as in (D). Arrowheads indicate lymphocytic infiltration (magnification, 200x). F. Serum alanine aminotransaminase activity in the HBcAg- or mock-immunized C57BL/6 mice after hydrodynamic injection.

The immune response to the HBV nucleocapsid Ag is believed to play a critical role in the control of HBV infection. HBc-specific CD8^+^ T cells contribute to viral clearance by killing infected cells (cytolytic HBV control) and/or inhibiting viral replication by producing cytokines such as IFN-γ and TNF-α (noncytolytic HBV control) [4], [5], [22]. Next, we aimed to demonstrate the usefulness of our assay system for monitoring HBc-specific immunity in the liver after vaccination. Naïve C57BL/6 mice were immunized with pVAX1-HBc or pVAX1 thrice intramuscularly within a 2-week interval and subsequently given a hydrodynamic injection of pGL3/Fluc-HBV1.2. After being challenged with pVAX1-HBc, a vigorous antibody response to HBV core protein was observed in C57BL/6 mice (Fig. S3). Secretion of HBsAg and HBV DNA into the blood were monitored over time (Fig. 2A and B). The levels of serum HBsAg and HBV DNA in HBcAg-immunized mice were similar to those in the mock-immunized mice, from the 1 day to the 14th day after hydrodynamic injection. At later time points, the serum HBsAg and HBV DNA levels in the HBcAg-immunized mice declined rapidly and were undetectable at 28 dpi but remained high in the mock-immunized mice. Next, we used in vivo imaging to characterize the real-time kinetics of clearance of hepatocytes expressing the HBV genome following hydrodynamic injection (Fig. 2C). Fig. 2C clearly shows that only HBcAg-immunized mice, but not mock-immunized mice, controlled luciferase expression in the liver. Longitudinal measurements of bioluminescence demonstrated that vaccinated animals had cleared most of the HBV-Fluc co-expressing hepatocytes between 14 dpi and 28 dpi, whereas mock-treated animals failed to clear the transfected hepatocytes. Therefore, we tried to address whether HBcAg-specific immunity is associated with HBV clearance in C57BL/6 mice. We examined the frequency of CTLs and HBcAg-specific IFNγ-producing CTLs in the splenocytes and hepatocytes of HBcAg- or mock-immunized C57BL/6 mice at 21 dpi using a FACS assay (Fig. 2D). High frequencies of HBcAg-specific IFNγ-producing CTLs were detected both in the splenocytes and hepatocytes of HBcAg-immunized mice, indicating greater HBcAg-specific immunity induced by pVAX1-HBc vaccination. Scattered infiltrating CTLs were also observed in HBcAg-immunized mice, but livers of mock-immunized mice showed no obvious inflammatory response (Fig. 2E). To further characterize liver tissue toxicity during the antigen clearance process, ALT values at various time points were also measured (Fig. 2F). There was a transient and rapid increase in the serum ALT values immediately after the hydrodynamic injection in both mice, but these values approached the normal range at 4 dpi. We observed increased ALT serum levels only in HBcAg-immunized mice, but not in mock-immunized mice, at 14 dpi and 21 dpi. These findings suggest that infiltrating CTLs may exert effector functions against infected hepatocytes.

**Figure 3:**
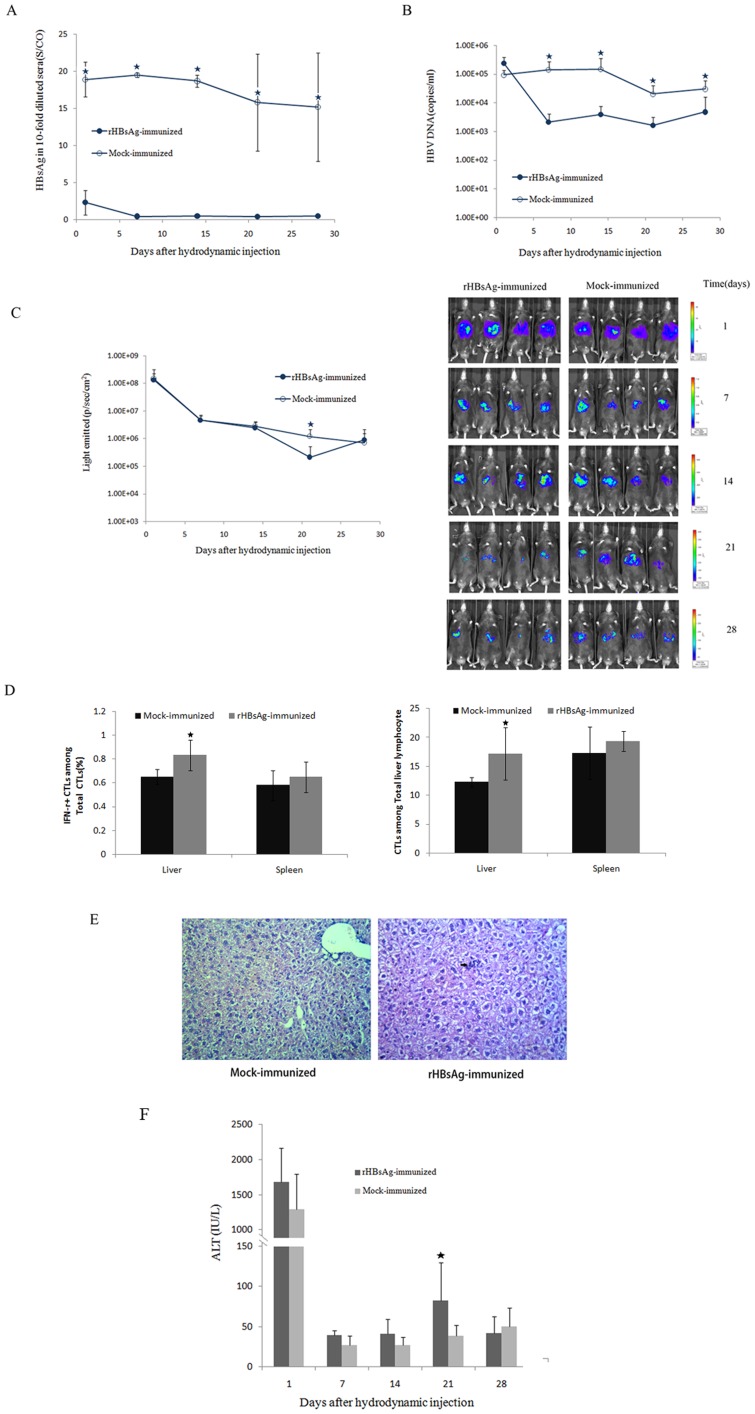
Monitoring HBs-specific humoral and cell-mediated immunity in the mice after immunization. A. Titers of serum HBsAg in the rHBsAg- or mock-immunized C57BL/6 mice after hydrodynamic injection of pGL3/Fluc-HBV1.2. B. Serum HBV DNA in the rHBsAg- or mock-immunized C57BL/6 mice after hydrodynamic injection. C. Real-time in vivo imaging of Fluc expression in the rHBsAg- or mock-immunized C57BL/6 mice over a period of 28 days after hydrodynamic injection. D. Liver- and spleen-associated CTLs were stimulated ex vivo with HBV peptides for 5 hours. *Left*: percentages of interferon-γ–secreting CTLs on day 21 postinfection (n = 4). *Right*: percentages of CTLs on day 21 postinfection (n = 4). Statistical differences are indicated as follows: * = p<0.05. E. H&E staining for infiltrates of inflammatory cells on day 21 (magnification, 200x). F. Serum alanine aminotransaminase activity in the rHBsAg- or mock-immunized C57BL/6 mice after hydrodynamic injection.

Currently, the commercial recombinant Hepatitis B surface antigen vaccine is widely used to prevent HBV infection by inducing effective humoral immunity [23]. This vaccine elicits relatively weak cell-mediated immune responses, particularly regarding the antigen-specific CTL response. Therefore, prior immunization with this vaccine is unable to facilitate clearance of the virus from infected cells. Here, we used our assay system to monitor HBs-specific humoral and cell-mediated immunity in mice after vaccination. Naïve C57BL/6 mice were immunized with rHBsAg or NaCl thrice subcutaneously in the back within a 2-week interval and subsequently given hydrodynamic injection of pGL3/Fluc-HBV1.2. After being challenged with rHBsAg, C57BL/6 mice had already developed protective levels of anti-HB antibodies (Fig. S4). Most of the mock-immunized C57BL/6 mice maintained antigenemia for both HBsAg and HBV DNA after pGL3/Fluc-HBV1.2 injection (Fig. 3A and B). However, all rHBsAg-immunized mice cleared HBsAg from their sera within 7 dpi (Fig. 3A). This finding demonstrates that strong antibody responses induced by rHBsAg protein succeeded in clearing the serum of HBsAg. Additionally, serum HBV DNA levels started to decline after the clearance of HBsAg in the rHBsAg-immunized mice but continued to remain at a high level (>1×10^3^ copies/ml) at all time points (Fig. 3B). Next, bioluminescence imaging was performed to examine Fluc activity. The result showed that luciferase activity was lower in rHBsAg-immunized mice than that in mock-immunized mice at 21 dpi (Fig. 3C). The frequency of CTLs and HBsAg-specific IFNγ-producing cells in the spleens and livers of C57BL/6 mice at 21 dpi was examined. Greater numbers of CTLs and HBsAg-specific IFNγ-producing CTLs were detected only in the hepatocytes from HBsAg-immunized mice (Fig. 3D). Although we observed a small yet significant increase in HBV-specific CTLs in the livers of the rHBsAg-immunized mice at 21dpi, immunization apparently failed to clear the HBV infection from the transfected hepatocytes. H&E staining showed small infiltrates of inflammatory cells (Fig. 3E). We also observed slightly increased ALT serum levels in HBsAg-immunized mice at 21 dpi (Fig. 3F).

## Discussion

Previously, bioluminescence imaging was successfully used to unravel important pathophysiological mechanisms of infection and persistence of various microorganisms [24]–[26]. Here, we employed bioluminescence detection of luciferase expression in HBV-expressed hepatocytes to follow HBV protein and DNA levels in these cells. In our animal model, a single hydrodynamic injection of a replication-competent HBV DNA, pGL3/Fluc-HBV1.2, into mice resulted in HBV persistence for more than 15 weeks in a significant proportion of recipients. Huang and colleagues have shown that HBV persistence is determined by the mouse genetic background and plasmid backbone [11]. Their group found that the AAV vector favors long-term transgene expression in hepatocytes, whereas injection of pGEM4Z/HBV1.2 (in which the HBV DNA sequence from pAAV/HBV1.2 was cloned into pGEM4Z) into mice produced only transient antigenemia. We injected pGL3/Fluc-HBV1.2, in which the pGL3 vector was substituted for the AAV vector, into mice also produced persistent antigenemia. This result showed that the pGL3 vector can also facilitate HBV persistence in the mouse liver. We also determined that the mouse genetic background can affect HBV persistence. Ten micrograms of pGL3/Fluc-HBV1.2 DNA was injected hydrodynamically into the tail veins of male C57BL/6 (H-2b) or BALB/c (H-2d) mice. In C57BL/6 mice, the HBsAg levels declined much more slowly than those in BALB/c mice. Of the C57BL/6 mice, approximately 80% remained HBsAg-positive at 10 weeks postinjection. In contrast, only approximately 40% of the BALB/c mice remained HBsAg-positive at 10 weeks postinjection. The serum HBV DNA levels in C57BL/6 mice were similar to those in the BALB/c mice. Fluc expression in the livers of all of the mice was sustained for many days at a stable level. But Fluc activity in the C57BL/6 mice was one order of magnitude lower than that in BALB/c mice. It is thought that this difference is partly attributable to the black hair of the C57BL/6 mice absorbing the light.

It is now believed that HBV-specific CD8^+^ T cells play a critical role in the control of HBV replication and in the pathogenesis of the disease via the destruction of infected liver cells. HBV core antigen (HBcAg) is recognized as the most efficient agent that primes human leucocyte antigen (HLA) class II-restricted CTL responses, which correlate with viral clearance in acute and self-limiting HBV patients; furthermore, the adoptive transfer of HBcAg-reactive T cells is associated with the resolution of chronic HBV infection [27]–[29]. In the present study, we employed bioluminescence detection of luciferase expression in HBV-infected hepatocytes to measure HBcAg-specific immune responses directed against these infected hepatocytes. Our data demonstrated that Fluc expression in the livers of the majority of HBcAg-immunized mice gradually became undetectable, accompanied by the rapid clearance of serum HBsAg and HBV DNA within 28 days after hydrodynamic injection of pGL3/Fluc-HBV1.2. Most of the mock-immunized mice maintained antigenemia for both HBsAg and HBV DNA, and additionally, these mice also sustained Fluc expression in the liver. The HBc-specific humoral and cellular immune responses were further analyzed. The results demonstrated that HBc DNA-based vaccination induced both strong antigen-specific T cell and high titer antibody responses systematically and in the liver. Furthermore, immunized mice showed strong cytotoxic responses that eliminated the HBV transfected hepatocytes.These responses were consistent with an increase in ALT and lymphocyte infiltration in the liver, visualized by H&E staining of liver tissue sections. Altogether, our data indicated that CTL-induced killing of HBV-infected hepatocytes occurred and that this process could be detected in vivo by bioluminescence imaging of luciferase expression, a similiar procedure developed by Stabenow et al. for studying T cell-mediated immunity in the liver using adenoviral transfer system [12]. But adenoviral system needs transfection, virus packaging, virus amplification and purification. Thus our hydrodynamic injection model is simpler and more accessible than adenoviral system.

We also used our assay system to monitor rHBsAg-mediated immunity in the mice after vaccination. Our data suggested that these recombinant S protein elicited protective HBs-specific antibody responses and a weak T-cell immune responses in the vaccinated mice. The HBs-specific humoral-mediated immunity could clear serum HBsAg and reduce HBV DNA levels. But the weak HBsAg-specific CTL response at 21dpi apparently failed to clear the transfected hepatocytes.

In conclusion, the present study demonstrates that HBV clearance can be detected in vivo by bioluminescence imaging of reporter gene expression. Direct *in vivo* determination of HBV clearance provides a new tool to characterize the principles underlying antiviral immunity in the liver. This assay system will become a powerful tool for the study of HBV-specific immunity in the liver.

## Supporting Information

Figure S1 Schematic diagram of plasmids pGL3 -HBV1.2 and pGL3/Fluc-HBV1.2.
(TIF)

Figure S2 HBV gene and Fluc expression in Huh7 cells. A. Titer of HBsAg in the supernant of pGL3 -HBV1.2, pGL3-CP-Fluc and pGL3/Fluc-HBV1.2 transfected Huh-7 cell. B. Fluc expression was detected by Luciferase Assay.
(TIF)

Figure S3 Serum anti-HBc in immune C57BL/6 mice before and 21 days after hydrodynamic injection of HBV.
(TIF)

Figure S4 Serum anti-HBs in immune C57BL/6 mice before and 21 days after hydrodynamic injection of HBV.
(TIF)
