# Supplementary figures and images for: A Mouse Model for Studying the Clearance of Hepatitis B Virus In Vivo Using a Luciferase Reporter
Source: PLoS One. 2013 Apr 5;8(4):e60005. doi: 10.1371/journal.pone.0060005 (PMC3618179; doi:10.1371/journal.pone.0060005)

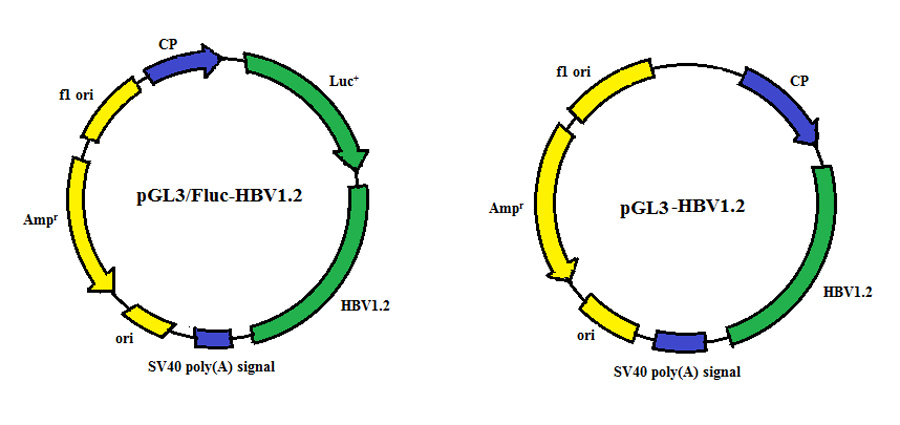

Supplement: Figure S1 — Schematic diagram of plasmids pGL3 -HBV1.2 and pGL3/Fluc-HBV1.2. (TIF) [file pone.0060005.s001.tif]

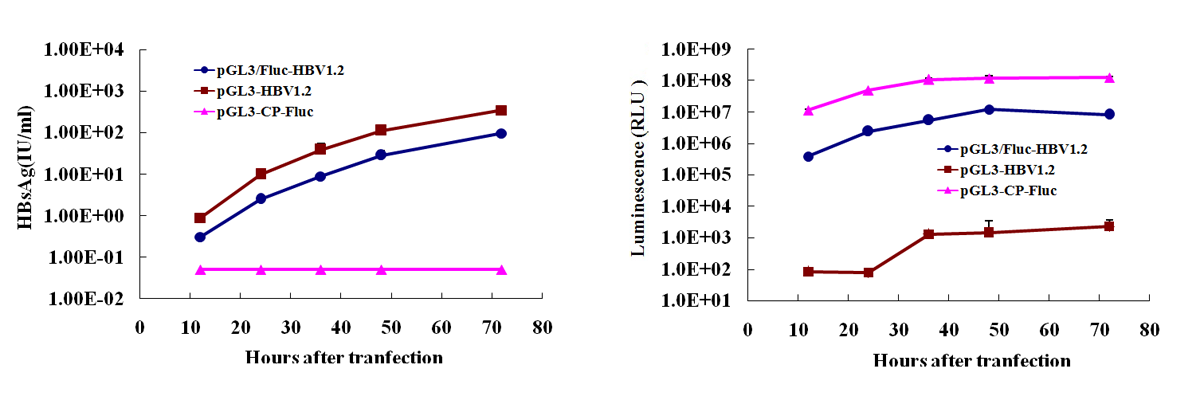

Supplement: Figure S2 — HBV gene and Fluc expression in Huh7 cells. A. Titer of HBsAg in the supernant of pGL3 -HBV1.2, pGL3-CP-Fluc and pGL3/Fluc-HBV1.2 transfected Huh-7 cell. B. Fluc expression was detected by Luciferase Assay. (TIF) [file pone.0060005.s002.tif]

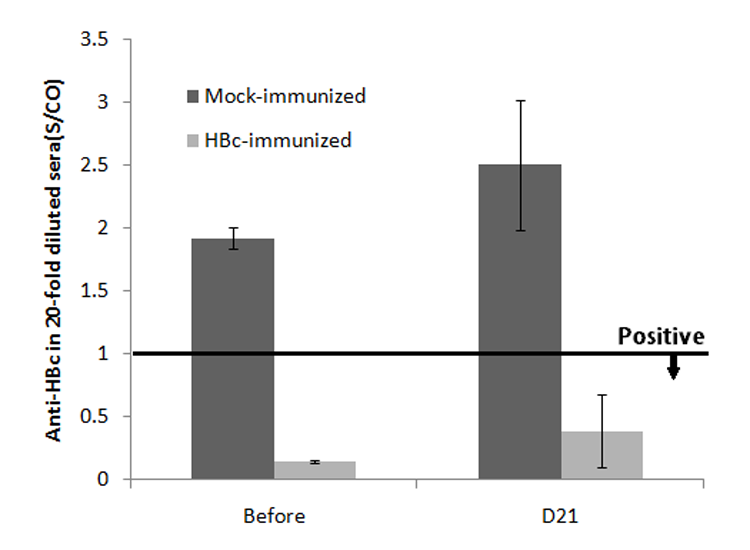

Supplement: Figure S3 — Serum anti-HBc in immune C57BL/6 mice before and 21 days after hydrodynamic injection of HBV. (TIF) [file pone.0060005.s003.tif]

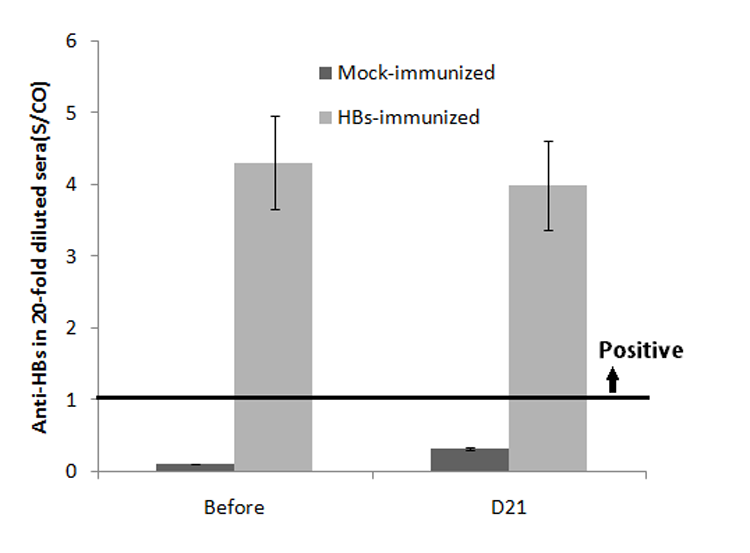

Supplement: Figure S4 — Serum anti-HBs in immune C57BL/6 mice before and 21 days after hydrodynamic injection of HBV. (TIF) [file pone.0060005.s004.tif]
